# Chromoblastomycosis: a case series from Sumba, eastern Indonesia

**DOI:** 10.1093/ced/llaf111

**Published:** 2025-03-08

**Authors:** Gladys O Siregar, Maria Harianja, Hanggoro T Rinonce, Eddy Zulfikar, Claus Bøgh, Messe R Ataupah, Ruth D Laiskodat, Evivana S Sundari, Hardyanto Soebono, Marlous L Grijsen

**Affiliations:** Sumba Foundation, Sumba, Indonesia; Sumba Foundation, Sumba, Indonesia; Department of Anatomical Pathology, Faculty of Medicine, Public Health and Nursing, Universitas Gadjah Mada, Yogyakarta, Indonesia; Department of Anatomical Pathology, Dr. Sardjito Hospital, Yogyakarta, Indonesia; Sumba Foundation, Sumba, Indonesia; Sumba Foundation, Sumba, Indonesia; Province Health Office, East Nusa Tenggara, Kupang, Indonesia; Province Health Office, East Nusa Tenggara, Kupang, Indonesia; Department of Dermatology and Venereology, Siloam Hospital, Kupang, Indonesia; Department of Dermatology and Venereology, Universitas Gadjah Mada, Yogyakarta, Indonesia; Center for Tropical Medicine, Faculty of Medicine, Public Health and Nursing, Universitas Gadjah Mada, Yogyakarta, Indonesia; Oxford University Clinical Research Unit Indonesia, Faculty of Medicine Universitas Indonesia, Jakarta, Indonesia; Centre for Tropical Medicine and Global Health, Nuffield Department of Medicine, University of Oxford, Oxford,UK

## Abstract

Chromoblastomycosis is a highly neglected implantation mycosis, typically affecting agricultural workers in impoverished rural communities in (sub)tropical regions. We present a case series of eight individuals with chromoblastomycosis that were identified through teledermatology in Sumba, a remote island in eastern Indonesia. We highlight the challenges faced in managing complex diseases like chromoblastomycosis in underserved communities and illustrate the value of telemedicine in empowering frontline healthcare workers and improving access to skin care.

Dear Editor, Chromoblastomycosis (CBM) is an implantation mycosis caused by pigmented fungi like *Fonsecaea*, *Phialophora* or *Cladophialophora* spp. that are widely found in nature.^1^ CBM typically affects agricultural workers following transcutaneous inoculation from micro or macro trauma during their daily activities, particularly in impoverished rural communities in Africa and South America.^1^ CBM is a neglected tropical skin disease (skin-NTD) of which the global disease burden is unknown. A recent literature review revealed that over the past century there were 7740 cases of individuals with CBM reported globally, with 1390 (18%) occurring in Asia (predominantly in China, Japan and India); 13 of these were in Indonesia.^1^

CBM is usually characterized by extensive verrucous nodular lesions, mainly affecting the lower limbs, which leads to disfigurement and is often associated with stigma, inability to work and a reduced quality of life.^1^ Treatment is difficult, with a high risk of recurrence, especially in late-stage diagnoses.^2^ Oral antifungals should be given for at least 6 months and can be combined with surgical debridement, cryotherapy, local heat application or adjunctive immunotherapy, such as topical imiquimod, which is a Toll-like receptor-7 agonist.^2^ However, ensuring consistent access to affordable treatment remains a major challenge in many resource-limited settings.^3^

This paper describes eight individuals affected by CBM that were identified through teledermatology in Sumba, a remote island in eastern Indonesia. In addition, we reviewed the literature for published cases of individuals with CBM in Indonesia during the past 15 years.

Sumba Foundation, a nongovernmental organization, runs five community health clinics that offer essential services free of charge in Sumba. In October 2020, we introduced a teledermatology service to assist frontline healthcare workers (HCWs) and provide training in the clinical and laboratory management of common and neglected skin diseases; this included training in direct microscopy as a rapid, reliable, cost-effective diagnostic tool for CBM, reducing the reliance on resource-intensive skin biopsies. Further details about this programme have been described elsewhere.^4^

Between 2021 and 2024, we identified eight individuals with CBM: seven men and one woman with a median age of 53 years (interquartile range 44–56). In all individuals, the lower extremities were affected (Figure 1a–h), involving the foot, ankle or lower leg; in one person the lesion extended up to the thigh. Three individuals recalled a history of trauma.

**Figure 1.**
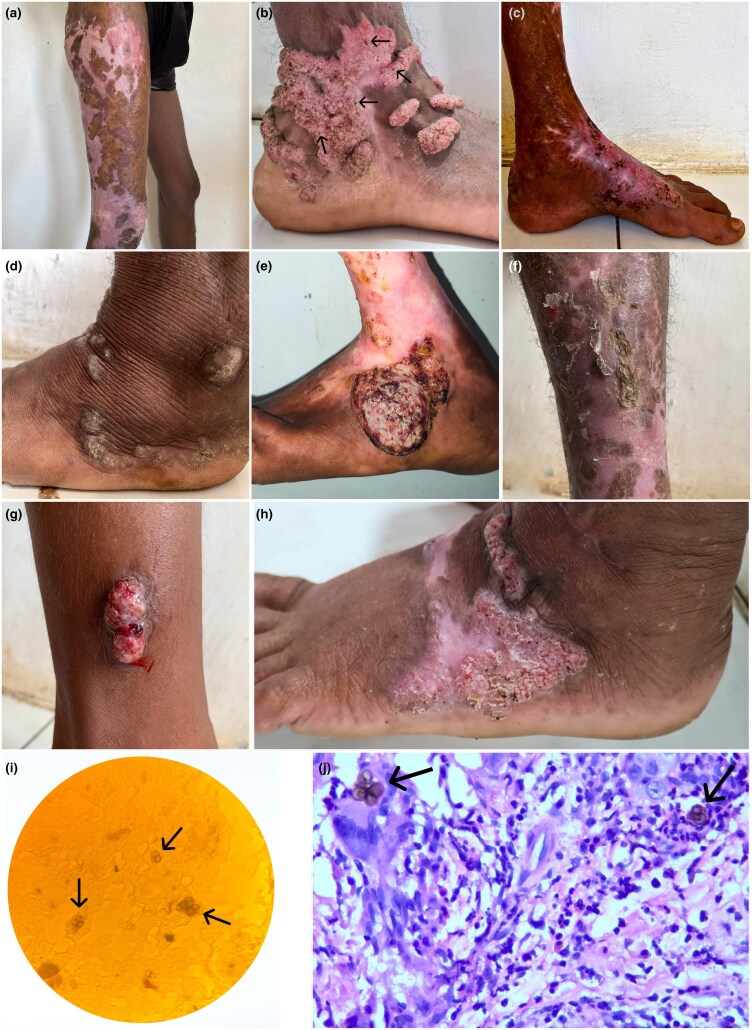
Chromoblastomycosis. (a–h) Clinical manifestations on the lower extremities of eight individuals diagnosed with chromoblastomycosis in Sumba, eastern Indonesia. (b) The dark spots visible on the lesion’s surface (indicated by the arrows) represent optimal sites for skin scrapings, increasing the likelihood of detecting fungal cells (e) Squamous cell carcinoma as a sign of long-standing infection. (i) Direct microscopy using potassium hydroxide wet mount showing the characteristic sclerotic bodies (also referred to as ‘medlar bodies’ or ‘copper pennies’), which are globe-shaped, golden brown pigmented, thick-walled single or multicellular clusters of fungal cells that often have a transverse septum (arrow; immersion oil × 100 magnification). (j) Haematoxylin and eosin stain showing sclerotic bodies (arrow) inside a multinucleated giant cell accompanied by neutrophils and eosinophils (× 40 magnification). Images retrieved via teledermatology using smartphones.

All individuals were HIV-negative; four had a poor nutrition status with a body mass index below 18.5 kg m^–2^. The duration of symptoms ranged from 6 months to more than 20 years. Three individuals faced challenges walking and were no longer able to cultivate their land. All individuals had received multiple treatments in the past, including oral corticosteroids under the differential diagnosis of lichen simplex chronicus or psoriasis.

The diagnosis of CBM was confirmed with a skin scraping through direct microscopy with 10% potassium hydroxide and histopathology illustrating the characteristic pigmented fungal cells (Figure 1i, j). Fungal cultures were not feasible in our setting.

All individuals initiated treatment with oral ketoconazole 200–400 mg daily, as alternative, more effective drugs, such as itraconazole, were not available in our setting. Two individuals have shown moderate improvement after taking ketoconazole for almost 2 years. The remaining individuals only recently started antifungal treatment. One person with a long-standing infection was diagnosed with histopathology-confirmed squamous cell carcinoma (Figure 1e), which ultimately led to the amputation of his lower leg.

This is, to the best of our knowledge, the first report describing the prevalence of CBM on Sumba Island. Between 2009 and 2024, around 40 other cases of individuals with presumed CBM have been reported across Indonesia (Table S1; see Supporting Information), showcasing that CBM is endemic in the region. The available data highlight the challenges faced in managing complex diseases like CBM in low- and middle-income countries. In most endemic settings, patients are often treated by frontline HCWs, who often lack the specific skills and resources for diagnosing and managing skin-NTD.

The diverse presentation of CBM, which can manifest as verrucous, nodular, tumoral, plaque-like, atrophic or ulcerative lesions, poses a significant diagnostic challenge, frequently leading to misdiagnosis, as observed in our case series. Prolonged duration of CBM may also be complicated by secondary bacterial infection, lymphoedema, tissue fibrosis, contractures and squamous cell carcinoma.^1^ Diagnostic delays, low health literacy, limited treatment options and poor access to affordable effective medication coupled with adherence challenges, which are associated with prolonged treatment, contribute to advanced disease and poor health outcomes.

Treatment guidelines for CBM are currently lacking. Itraconazole and terbinafine are recommended as first-line treatments,^1^ although these drugs are costly and not widely available in many endemic settings,^3^ especially considering the need for prolonged treatment, often up to several years. Rural areas in eastern Indonesia, largely dependent on the government healthcare system, feature the lowest level of drug availability in the country.^5^ Consistent access to essential medication is the key determining step for successful treatment outcomes.

Our teledermatology platform demonstrated that telemedicine empowers frontline HCWs in recognizing and diagnosing skin diseases and can substantially improve access to specialized healthcare for remote underserved populations. To achieve the goals in the World Health Organization NTD Roadmap 2021–2030, structural investments by government and related stakeholders are required to address the pressing needs related to CBM and other skin-NTDs.

## Supplementary Material

llaf111_Supplementary_Data

## Data Availability

The data underlying this article are available upon request from the corresponding author.
