# Supplementary material for: Chromoblastomycosis: a case series from Sumba, eastern Indonesia
Source: Clin Exp Dermatol. 2025 Mar 8;50(7):1447–50. doi: 10.1093/ced/llaf111 (PMC12188220; doi:10.1093/ced/llaf111)
Supplement: llaf111_Supplementary_Data [file llaf111_supplementary_data.docx]

**Table S1** Summary of cases of individuals with presumed chromoblastomycosis reported in Indonesia between 2009 and 2024 (*n* = 48)^‡^

| **First author** | **Number of patients** | **Location in Indonesia** | **Sex** | **Age (years)** | **Duration of complaints (years)** | **Occupation** | **Diagnostic test (KOH, skin biopsy)** | **Fungal culture** | **Treatment (dosage) and duration** |
| --- | --- | --- | --- | --- | --- | --- | --- | --- | --- |
| **Rosvanti (2009)^1^** | 1 | East Java | M | 46 | 1 | Driver | Biopsy | *F. pedrosoi* | Itraconazole (100 mg BID) for 9 months |
| **Haykal (2013)^2^**^∞^ | 1 | - | - | - | - | - | - | *Phialophora verrucosa* |  |
| **Lasut (2015)^3^** | 1 | North Sulawesi | F | 37 | 20 | Farmer | KOH and biopsy | *F. pedrosoi* | Itraconazole (200 mg BID) for at least 2 months |
| **Sukmawati (2015)^4^** | 6 | East Java (5),  North Kalimantan (1) | - | - | - | Farmers (n = 3), Taylor (1), Private sector (1), Civil servant (1) | Biopsy | *F. pedrosoi* (n = 3), *F. dermatitidis* (1), *F. verucosa* (1) | Ketoconazole (n = 5), itraconazole (1, dosage and duration not reported) |
| **Yahya (2016)^5∆^** | 3 | East Java | - | 47 | - | - | KOH and biopsy | *Phialophora sp.*(n = 1) | Itraconazole for 3 months,  followed by itraconzole + terbinafine  (dosage and total duration not reported) |
| **Rosvanti (2017)^6^** | 1 | East Java | M | 33 | 15 | Gardener | KOH and biopsy | *F. pedrosoi* | Ketoconazole (200 mg BID) for 9 months, followed by itraconazole (200 mg BID) + terbinafine (250 mg BID) for 8 months |
| **Khairani (2021)^7^** | 1 | South Sumatera | M | 11 | 5 | Student | Biopsy | - | Itraconazole (100 mg OD) for 12 months |
| **Dharmawan (2021)^8^** | 1 | Central Java | M | 70 | 6 | Farmer | KOH and biopsy | *F. pedrosoi* | Itraconazole (400 mg/day) for 1 week per month for at least 3 months combined with cryotherapy once per week |
| **Horo (2022)^9^** | 1 | Bali | F | 24 | 0.5 | Student | - | *Cladophialophora carrionii* | Itraconazole(400 mg/day) for 1 week per month for at least 3 months |
| **Indranarum (2023)^10^** | 1 | East Java | M | 47 | 17 | Construction worker | KOH and biopsy | *F. pedrosoi* | Itraconazole (200 mg BID) + local heat therapy + CO_2_ laser (at least 19 weeks);  patient was still on treatment and monitoring |
| **Topik (2023)^11^** | 1 | Aceh | M | 51 | 0.6 | Construction worker | Biopsy | - | Itraconazole (200 mg BID);  duration not mentioned;  patient was still on treatment and monitoring |
| **Ariani (2023)^12^** | 5 | West Sumatra | 1 F,  4 M | 57 (54–60)^◊^ | 15 (15–20)^◊^ | Farmers | KOH and biopsy | *F. pedrosi* | Itraconazole 400 mg/day 1 week per month for a total of 3 months;  patients were still on treatment and monitoring |
| **Handayani (2023)^13^** | 1 | Aceh | M | 72 | 1 | - | Biopsy | - | Itraconazole (200 mg BID),  duration not mentioned;  patient patients were still on treatment |
| **Earlia (2024)^14^** | 1 | Aceh | F | 66 | 18 | Farmer | Biopsy | - | Itraconazole (100 mg BID) for 8 months;  patient was still on treatment |
| **Pramita (2024)^15^**^∞^ | 15 | Bali | - | - | - | - | - | - | - |
| **Siregar (2025, this article)** | 8 | Sumba | 1 F,  7 M | 53 (44–56)^¥^ | 10 (5–17)^◊^ | Farmers | KOH and biopsy | - | Ketoconazole (200–400 mg/day) for at least 24 months (n = 2), one patient required an amputation due to squamous cell carcinoma, remaining 5 individuals started ketoconazole recently |

^‡^ This number includes the eight individuals that we recently diagnosed with chromoblastomycosis in Sumba Island. ^∞^ This report only provided the total number of individuals diagnosed with chromoblastomycosis, no further details were provided. ^∆^ 3 persons with chromoblastomycosis were reported but only one was explained in detail. ^◊^ The median plus interquartile range (IQR) are reported. ^¥^ One individual was aged 16 years old.

1. Rosvanti A, Andriyani C, Lumintang H, Suyoso S. Kromoblastomikosis yang Disebabkan oleh Fonsecaea pedrosoi. Periodical of Dermatol and Venereol. 2009;21(2): 180–184.

2. Haykal Ahmad, Kadir Dirmawati, Amin Safruddin. Chromoblastomycosis et causaphialophora verrucosa. Indonesian Journal of Dermatology and Venereology. 2013;1(4):66–72.

3. Lasut MV, Tanamal RS, Kapantow GM. Kasus Kromoblastomikosis pada Seorang Perempuan. Jurnal Biomedik. 2015;7(1):62069.

4. Sukmawati N, Ervianty E. Characteristic of Subcutan Mycosis: A Retrospective study. Berkala Ilmu Kesehatan Kulit dan Kelamin 2015;27(3):183–190.

5. Yahya S, Widaty S, Miranda E, Bramono K, Islami AW. Subcutaneous mycosis at the Department of Dermatology and Venereology dr. Cipto Mangunkusumo National Hospital, Jakarta, 1989–2013. J Gen Pro DVI. 2016;1(2):36–43.

6. Rosyanti A, Suyoso S. Chromomycosis Treatment with Combination of Itraconazole and Terbinafine. Periodical of Dermatol Venereol. 2017;8(2):168–174.

7. Khairani FA, Pamela RD. A Rare Case of Chromoblastomycosis Resembling Keloid in an Indonesian Child. Cureus. 2021;13(10): e18490.

8. Dharmawan N, Fiqri A. Chromoblastomycosis Treatment with Combination Therapy of Itraconazole (Pulse Dose) and Cryotherapy. Jurnal Profesi Medika: Jurnal Kedokteran Dan Kesehatan. 2023;15(2).

9. Horo R., Rusyati LMM, Karna NLPRV, Sari NPARY. Kasus kromoblastomikosis yang awalnya diduga furunkel pada seorang perempuan. Intisari Sains Medis. 2022;13(1): 362–366.

10. Indranarum T, Axelia PG, Sandhika W, Listiawan MY, Ervianti E. A case of chromoblastomycosis caused by Fonsecaea pedrosoi: challenge in diagnosis. Bali Medical Journal. 2023;12(3): 3407–3410.

11. . Topik MM and Faizah S. Diagnosis dan Rencana Tatalaksana Chromoblastomycosis. Jurnal Ventilator 2023;1(3):30–38.

12. Ariani T, Rizal Y, Veroci RL. Clinical and Mycological Spectrum of Chromoblastomycosis: a Case Series. J Pak Assoc Dermatol.2023 [cited 2024Jul.16];33(4):1750–61.

13. Handayani M and Topik MM. Case Report: Chromoblastomycosis. Diponegoro Medical Journal 2023;12(6):411–415.

14. Earlia N, Maulida, M, Handriani R, Kamarlis RK, Pradistha A. Chronic cutaneous chromoblastomycosis: A rare case. J Gen Proced Dermatol Venereol Indones. 2024;8(1):40–45. DOI: 10.7454/jdvi.v8i1.1183

15. Pramita IGAR, Karna NLPRV, Karmila IGAAD. Characteristic of Subcutaneous Mycosis Patients at Prof. Dr. I.G.N.G Hospital, Denpasar, Bali: An Observational Retrospective Study. International Journal of Scientific Advances 2024;5(2):246–248.
